# Lack of Benefit of Adjusting Adaptively Daily Invitations for the Evaluation of the Quality of Anesthesiologists’ Supervision and Nurse Anesthetists’ Work Habits

**DOI:** 10.7759/cureus.49661

**Published:** 2023-11-29

**Authors:** Franklin Dexter, Bradley J Hindman, Kokila Thenuwara

**Affiliations:** 1 Anesthesia, University of Iowa, Iowa City, USA

**Keywords:** professional practice gaps, nurse anesthetists, anesthesiologists, ongoing professional practice evaluation, bernoulli cumulative sum, work habits, clinical supervision, evaluation of performance

## Abstract

Introduction

Whenever a department implements the evaluation of professionals, a reasonable operational goal is to request as few evaluations as possible. In anesthesiology, evaluations of anesthesiologists (by trainees) and nurse anesthetists (by anesthesiologists) with valid and psychometrically reliable scales have been made by requesting daily evaluations of the ratee’s performance on the immediately preceding day. However, some trainees or nurse anesthetists are paired with the same anesthesiologist for multiple days of the same week. Multiple evaluations from the same rater during a given week may contribute little incremental information versus one evaluation from that rater for the week. We address whether daily evaluation requests could be adjusted adaptively to be made once per week, hopefully substantively reducing the number of evaluation requests.

Methods

Every day since 1 July 2013 at the studied department, anesthesia residents and fellows have been requested by email to evaluate anesthesiologists’ quality of supervision provided during the preceding day using the De Oliveira Filho supervision scale. Every day since 29 March 2015, the anesthesiologists have been requested by email to evaluate the work habits of the nurse anesthetists during the preceding day. Both types of evaluations were made for interactions throughout the workday together, not for individual cases. The criterion for an electronic request to be sent is that the pair worked together for at least one hour that day. The current study was performed using evaluations of anesthesiologists' supervision and nurse anesthetists' work habits through 30 June 2023.

Results

If every evaluation request were completed by trainees on the same day it was requested, trainees would have received 13.5% fewer requests to evaluate anesthesiologists (9367/69,420), the maximum possible reduction. If anesthesiologists were to do the same for their evaluations of nurse anesthetists, the maximum possible reduction would be 7.1% fewer requests (4794/67,274). However, because most evaluations were completed after the day of the request (71%, 96,451/136,694), there would be fewer requests only if the evaluation were completed before or on the day of the next pairing. Consequently, in actual practice, there would have been only 2.4% fewer evaluation requests to trainees and 1.5% fewer to anesthesiologists, both decreases being significantly less than 5% (both adjusted P <0.0001). Among the trainees’ evaluations of faculty anesthesiologists, there were 1.4% with very low scores, specifically, a mean score of less than three out of four (708/41,778). Using Bernoulli cumulative sum (CUSUM) among successive evaluations, 72 flags were raised over the 10 years. Among those, there were 36% with more than one rater giving an exceptionally low score during the same week (26/72). There were 97% (70/72) with at least one rater contributing more than one score to the recent cumulative sum.

Conclusion

Conceptually, evaluation requests could be skipped if a rater has already evaluated the ratee that week during an earlier day working together. Our results show that the opportunity for reductions in evaluation requests is significantly less than 5%. There may also be impaired monitoring for the detection of sudden major decreases in ratee performance. Thus, the simpler strategy of requesting evaluations daily after working together is warranted.

## Introduction

Whenever a department implements the evaluation of professionals, a reasonable operational goal is to request as few evaluations as possible [1]. This is because individuals who perceive heavy workloads in performing performance reviews report they feel they are less productive [1].

In anesthesiology, evaluations of anesthesiologists (by trainees) and nurse anesthetists (by anesthesiologists) with valid and psychometrically reliable scales have been made by requesting daily evaluations of the ratee’s performance on the immediately preceding day [2,3]. However, conceptually, a trainee or nurse anesthetist could be paired with the same anesthesiologist for all five weekdays. The trainee’s evaluations of the anesthesiologist, or the anesthesiologist’s evaluations of the nurse anesthetist, on multiple days of the same week, may contribute little incremental information versus one evaluation for the week [2,4,5]. Therefore, the question we address is whether daily evaluation requests could be adjusted adaptively to be made once per week, hopefully substantively reducing the number of evaluation requests. This means that rather than requesting evaluations from anesthesiologists each day who worked with a nurse anesthetist, and from trainees daily who worked with an anesthesiologist, we could skip extra requests for the same pairing for that week if an evaluation has already been completed. We examined if, by so doing, a substantive ($\geq$5%) reduction in evaluation requests could be realized. Our department is uniquely suited to answering this question because these daily evaluation programs have been ongoing for many years. As a secondary question, we explored whether implementation of no more than one request per week would have reduced information obtained from Bernoulli cumulative sum (CUSUM) analyses of occasions of several very low-performance scores in near succession [6].

## Materials and methods

The University of Iowa Institutional Review Board determined that this project (#202311086) does not meet the regulatory definition of human subjects research. None of the data used were patient data. All data used had blinded identifiers for raters and ratees.

Every day since the first day of July 2013 at the University of Iowa, every anesthesia resident and fellow has been requested by email to evaluate anesthesiologists’ quality of supervision provided during the preceding day using the De Oliveira Filho supervision scale [7,8]. Each of the nine items is scored: one never, two rarely, three frequently, or four always [2,5,7,8]. For example, “the faculty was promptly available to help me solve problems with patients and procedures” [2,5,7,8]. Anesthesiologist supervision scores provided by residents are negligibly different when the rated anesthesiologist has more American Society of Anesthesiologists' Relative Value Guide units of work that same day with other residents or nurse anesthetists (Kendall's tau = −0.057, standard error 0.014) [2]. Every day since 29 March 2015, the anesthesiologists have been requested by email to evaluate the work habits of the nurse anesthetists during the preceding day [4,9]. Each of the six items is scored on a five-point scale [4]. For example, a score of one was “consistently seemed unprepared for the case(s)” and a score of five was “consistently well prepared for case(s)” [4].

Both types of evaluations were made for interactions throughout the workday together, not for individual cases [2,4,5,9]. All items in evaluations had to be scored for an evaluation to be submitted. The criterion for sending an electronic request was that the pair worked together for at least one hour, counted using the Epic anesthesia data [2,4,5,9]. These were for interactions days or nights, workdays or weekends [2,4,5,9]. These were operating room and non-operating room time-based anesthetics [2,4,5,9]. Evaluation requests that were not completed in 14 days expired automatically. In another study, we found that seven-item evaluations were completed in less than one minute for 89% of evaluations and in less than two minutes for 96% of evaluations [10].

The current study was performed using evaluations of anesthesiologists’ supervision and nurse anesthetists’ work habits through the 30th day of June 2023, the date of the department’s most recent Ongoing Professional Practice Evaluation. Data fields used to investigate the primary question were service date, ratee in blinded format, rater in blinded format, and evaluation date if completed (Table 1).

**Table 1:** Raw counts of the data ^a^ Requests sent to trainees to evaluate anesthesiologists were 41% trainees in clinical anesthesia year one (n = 28,325), 32% in year two (n = 22,124), and 16% in year three (n = 11,384).

Statistics	Anesthesiologists’ Supervision	Nurse Anesthetists’ Work Habits
Counts of evaluation requests for an operating room or a non-operating room day pairing^a^	69,420	67,274
Distinct days with at least one evaluation request	3,522	2,911
Distinct raters	232	151
Distinct ratees	120	147
Distinct rater-ratee combinations with at least one evaluation request	12,339	9,099
Evaluations completed (%)	51,778 (75%)	56,599 (84%)
50^th^ percentile of days until evaluations are completed if done	3	2
25^th^ percentile of days until evaluations are completed if done	1	1
75^th^ percentile of days until evaluations are completed if done	6	5
Evaluation requests among raters receiving one evaluation request that day	46,004 (66%)	17,713 (26%)
Evaluation requests among raters receiving two evaluation requests that day	19,236 (28%)	18,940 (28%)
Evaluation requests among raters receiving three evaluation requests that day	3,657 (5%)	14,745 (22%)

Descriptive statistical methods were used to determine how many fewer evaluation requests would have been received and evaluations completed. This arithmetic was performed using two different methods. First, counts were made assuming, deliberately falsely, that all evaluations were completed on the same day they were requested. This approach was taken to learn the largest possible reduction in evaluation requests. Second, counts were made of evaluation requests wherein the first assignment pairing together for that week had been completed by the end of the day of the next occasion the pair worked together. The two-sided binomial test for a reduction in evaluations that would have been requested was compared with five percent, treating the Bonferroni-adjusted P <0.05 as significant. The Bonferroni adjustment was for the two comparisons. As explained in the Discussion, a reduction of at least 5% was considered sufficiently large to change workflow.

We also asked a secondary question: whether fewer evaluations from the same rater per week would functionally affect Bernoulli cumulative sum (CUSUM) daily calculations for detecting a sudden major decrease in ratee performance [6]. This is a confidential clinical quality control process to notify rapidly senior departmental management of potential concerns of very low scores. In brief, each night, an average supervision score of less than three on the four-point scale increases the cumulative sum [6]. An average work habit score of less than or equal to three on the five-point scale prompts an increase in the cumulative sum. Each supervision evaluation score of at least equal to three contributes to a decrease in the cumulative sum [6]. Each work habits score over three contributes to a decrease in the cumulative sum. If the cumulative sum exceeds a precalculated threshold, a confidential notification (a flag) is sent automatically to senior management, and the cumulative sum is reset [6]. The original article describing this process included an example of two raters in different operating rooms on the same day or week reported exceptionally low anesthesiologist performance [6]. The frequency of such pairings was significantly greater than expected by chance [6]. The probability of two separate raters independently raising such large concerns during the same week based on chance is vanishingly small [6]. Each flag prompts investigation to ensure the absence of other indications of a concerning change in the practitioner’s performance. We examined the prevalence of these reports from two or more raters.

## Results

If every evaluation request were completed by trainees on the same day it was requested, trainees would have received 13.5% fewer requests to evaluate anesthesiologists (Table 2). This was the maximum possible reduction. If anesthesiologists were to do the same for their evaluations of nurse anesthetists, the maximum possible reduction would have been 7.1% fewer requests. However, because most evaluations were completed after the day of the request (71%), there would be fewer requests only if the evaluation were completed before or on the day of the next pairing. Consequently, in actual practice, there would have been only 2.4% fewer evaluation requests to trainees and 1.5% fewer to anesthesiologists, both decreases being significantly less than 5% (both adjusted P <0.0001). The reduced workload of completing evaluations would have been comparable because 2.9% fewer evaluations would have been completed by trainees and 1.7% by anesthesiologists.

**Table 2:** Counts used in the results Evaluation requests in the third row did not change over time, with anesthesiologists' supervision Pearson correlation r = 0.015 and nurse anesthetists' work habits r = 0.011. Evaluations completed after the date of the request were overall 71%, where 71% = (54,034 + 42,417)/(69,420 + 67,274).

Statistics	Anesthesiologists’ Supervision	Nurse Anesthetists’ Work Habits
Counts of evaluation requests for operating room or non-operating room day pairing	69,420	67,274
Counts of evaluations completed	51,778	56,599
Fewer evaluation requests if every evaluation request were completed on the same day it was requested	9,367 (13.5%)	4,794 (7.1%)
Evaluations completed after the day of the request	54,034 (77.8%)	42,417 (63.1%)
Fewer evaluation requests were achievable in practice based on evaluations for the first pairing together having been completed by the end of the day on subsequent occasions together that week	1,635 (2.4%)	1,004 (1.5%)
Fewer evaluation completions that would have been achieved in practice based on dates of completions of earlier pairings together that week	1,510 (2.9%)	950 (1.7%)

Among the trainees’ evaluations of faculty anesthesiologists, 1.4% had very low scores, specifically, a mean score of less than three (Table 3). Using Bernoulli CUSUM among successive evaluations [6], 72 flags were raised over the 10 years. Among those, there were 36% with two or more raters giving an exceptionally low score during the same week. Among the anesthesiologists’ evaluations of nurse anesthetists, 1.0% had very low scores, specifically a mean work habit score of less than or equal to three. There were 33 flags raised over the eight-year three months. Among those, there were 9% with two or more raters giving a very low score during the same week. To quantify the impact of the disruption of this monitoring on the information provided to senior management [6], we used the 72 flags raised in trainees’ evaluations of faculty anesthesiologists (Table 3). There were 97% (70/72) with at least one rater contributing more than one score to the recent cumulative sum.

**Table 3:** Counts of the Bernoulli cumulative sum (CUSUM) monitoring ^a^ The flags were sent after a final very low score. Those very low scores were provided by a trainee in clinical anesthesia year one for 40% (n = 29), in clinical anesthesia year two for 35% (n = 25), and in year three for 22% (n = 16), similar to the distribution of evaluation requests (Table 1 footnote a).

Statistics	Anesthesiologists’ Supervision	Nurse Anesthetists’ Work Habits
Counts of evaluations completed	51,778	56,599
Evaluations with very low scores	708 (1.4%)	579 (1.0%)
Flags raised over the years of evaluations	72^a^	33
Flags with two or more raters giving a very low score during the same week	26 (36.1%)	3 (9.1%)

## Discussion

Anesthesia departments have several goals and responsibilities related to evaluations of clinical care. There are needs for ongoing professional practice evaluations to continue clinical privileges, there are requirements for promotion reviews and annual or semi-annual professional performance evaluations, and there should be a process to become aware of and promptly address acute changes in clinical performance [6]. These three needs are fully met by evaluating daily anesthesiologists’ quality of clinical supervision and nurse anesthetists’ work habits [2,4,5,9]. We examined whether the number of evaluations of anesthesiologists by trainees and nurse anesthetists by anesthesiologists could be substantively reduced by not requesting an evaluation when an evaluation has already been completed for the pairing during that week. The analyses show that, in practice, the reductions would be much less than 5%. The principal reason for the small benefit was that there were so many residents, faculty anesthesiologists, and nurse anesthetists that a given trainee-anesthesiologist pair and a given anesthesiologist-nurse anesthetist pair rarely work with each other more than once per week [3]. Thus, results will be different than for evaluations of large lectures [11]. Furthermore, if a change in the evaluation request process were made, there would be a substantive loss of information for senior management from the Bernoulli CUSUM process of detecting an acute change in performance [6]. Specifically, while 36% (26/72) of flags of anesthesiologists’ poor performance had two or more raters giving a very low score, 97% (70/72) had at least one rater contributing more than one very low score. Thus, if raters provided only one evaluation per week, many of the very low-performance flags would not have been created.

Extra hypothetical reasons support daily evaluation rather than not requesting another if the rater has already evaluated the ratee that week. First, if limited to one per week, the workload of completing evaluations could become highly unequal among department members. For example, anesthesiologists working in locations with many different nurse anesthetists would perform more evaluations weekly than anesthesiologists working in other locations. Second, suppose a faculty anesthesiologist worked with Resident A on Tuesday. On Friday, the faculty supervises Resident A in one operating room and Resident B in another. An incentive would be produced for the faculty to teach the anesthesiology Resident B more because the faculty would know that Resident A would not evaluate their performance. These conceptual reasons further suggest a daily evaluation system has strengths when using a brief, valid, and psychometrically reliable scale [4,5,7].

Our study was limited because the results apply to large departments with many anesthesiologists, resident physicians and fellows, and/or nurse anesthetists. There are many such departments with evaluations to be completed [12]. Smaller departments may attribute greater importance to confidential behavior reporting, such that Bernoulli CUSUM monitoring might be even more important, depending on daily evaluations [6]. On the other hand, smaller departments may have more personnel working together for the day more often than the studied department. Having learned that the principal reason for our results was that the anesthesiologists infrequently worked with the same trainee or nurse anesthetist more often than once per week, we repeated our literature search. We used PubMed on 16 November 2023: ( anesthesiologist*[TIAB] OR anaesthesiologist*[TIAB] OR anesthetist*[TIAB] OR anaesthetist*[TIAB] ) AND ( occasion*[TIAB] OR pair*[TIAB] OR together[TIAB] ) AND ("performance assessment*"[TIAB] OR "performance evaluation*"[TIAB] OR supervision[TIAB] OR "work habits"[TIAB] ). Among the 24 articles returned, four were relevant. Three of the four articles were earlier reports from our department assessing the reliability and validity of the supervision evaluations [2,8,13]. The fourth article reported that, in Germany, trainees often work with one anesthesiologist during the beginning of anesthesia residency [14]. That was true also for the studied department, consistent with the anesthesiologists and trainees having worked together more often than once per week for 13.5% of evaluation requests versus anesthesiologists and nurse anesthetists for 7.1% of requests, even though there were more trainees (232) than nurse anesthetists (147) (Table 1).

## Conclusions

Anesthesia departments can validly and reliably evaluate their anesthesiologists using a supervision scale and nurse anesthetists using a work habits scale. These are daily evaluations for operating rooms and non-operating room settings. Conceptually, evaluation requests could be skipped if the rater had already evaluated the ratee that week during an earlier day working together. Our results show that the opportunity for reductions in evaluation requests is significantly less than 5%. There may also be impaired monitoring for detecting sudden major decreases in ratee performance and associated information for senior management. Thus, the simple strategy of requesting evaluations daily after working together is warranted.

## References

[1] Oh Y (2023). Exploring the dysfunctional consequences of performance evaluation systems: how does ‘evaluation overload’ affect organizational performance?. Public Manag Rev.

[2] Dexter F, Ledolter J, Smith TC, Griffiths D, Hindman BJ (2014). Influence of provider type (nurse anesthetist or resident physician), staff assignments, and other covariates on daily evaluations of anesthesiologists' quality of supervision. Anesth Analg.

[3] Bayman EO, Dexter F, Ledolter J (2017). Mixed effects logistic regression modeling of daily evaluations of nurse anesthetists’ work habits adjusting for leniency of the rating anesthesiologists. Periop Care Oper Room Manag.

[4] Dexter F, Ledolter J, Hindman BJ (2017). Validity of using a work habits scale for the daily evaluation of nurse anesthetists' clinical performance while controlling for the leniencies of the rating anesthesiologists. J Clin Anesth.

[5] Dexter F, Ledolter J, Hindman BJ (2017). Measurement of faculty anesthesiologists' quality of clinical supervision has greater reliability when controlling for the leniency of the rating anesthesia resident: a retrospective cohort study. Can J Anaesth.

[6] Dexter F, Ledolter J, Hindman BJ (2014). Bernoulli cumulative sum (CUSUM) control charts for monitoring of anesthesiologists' performance in supervising anesthesia residents and nurse anesthetists. Anesth Analg.

[7] de Oliveira Filho GR, Dal Mago AJ, Garcia JH, Goldschmidt R (2008). An instrument designed for faculty supervision evaluation by anesthesia residents and its psychometric properties. Anesth Analg.

[8] Hindman BJ, Dexter F, Kreiter CD, Wachtel RE (2013). Determinants, associations, and psychometric properties of resident assessments of anesthesiologist operating room supervision. Anesth Analg.

[9] Dexter F, Bayman EO, Wong CA, Hindman BJ (2020). Reliability of ranking anesthesiologists and nurse anesthetists using leniency-adjusted clinical supervision and work habits scores. J Clin Anesth.

[10] O'Brien MK, Dexter F, Kreiter CD, Slater-Scott C, Hindman BJ (2019). Nurse anesthetists' evaluations of anesthesiologists' operating room performance are sensitive to anesthesiologists' years of postgraduate practice. J Clin Anesth.

[11] Kreiter CD, Lakshman V (2005). Investigating the use of sampling for maximising the efficiency of student-generated faculty teaching evaluations. Med Educ.

[12] Matava CT, Alam F, Kealey A, Bahrey LA, McCreath GA, Walsh CM (2023). The influence of resident and faculty gender on assessments in anesthesia competency-based medical education. Can J Anaesth.

[13] Dexter F, Hadlandsmyth K, Pearson AC, Hindman BJ (2020). Reliability and validity of performance evaluations of pain medicine clinical faculty by residents and fellows using a supervision scale. Anesth Analg.

[14] Goldmann K, Steinfeldt T, Wulf H (2006). Anaesthesia education at German university hospitals: the teachers' perspective - results of a nationwide survey [Article in German]. Anasthesiol Intensivmed Notfallmed Schmerzther.

